# Older Women in Australia: Facing the Challenges of Dual Sensory Loss

**DOI:** 10.3390/ijerph17010263

**Published:** 2019-12-30

**Authors:** Chyrisse Heine, Cathy Honge Gong, Susan Feldman, Colette Browning

**Affiliations:** 1 School of Allied Health, Human Services & Sport, College of Science, Health and Engineering, La Trobe University, Bundoora, VIC 3086, Australia; c.heine@latrobe.edu.au; 2 Centre for Research on Ageing, Health and Wellbeing, Research School of Population Health, College of Health and Medicine, Australian National University, Canberra, ACT 2617, Australia; 3 Australian Research Centre (ARC) Centre of Excellence in Population Ageing Research, Canberra, ACT 2601, Australia; 4 Independent Researcher, 41 Tyrone Street, South Yarra, VIC 3141, Australia; Susan@myaddress.net.au; 5 School of Nursing and Healthcare Professions, Federation University, Mt Helen Campus, Ballarat, VIC 3353, Australia; c.browning@federation.edu.au; 6 International Institute for Primary Health Care Research, Shenzhen 518000, China

**Keywords:** older women, dual sensory loss, physical health, mental health, social health, well-being, quality of life, Australia

## Abstract

With the increase in longevity, the number of women living into old age is rising and higher than that of men. Data was derived from the Melbourne Longitudinal Studies on Healthy Ageing Program, which included 533 women and 467 men aged 65 years and older, in Australia, over 10 years. Logistic regression modeling was used to investigate the prevalence of dual sensory loss and the unmet needs for vision and hearing devices in older women (compared to men) over time, as well as its impacts on self-reported general health, depression, perceived social activities, community service use and ageing in place. Results suggested that the prevalence of dual sensory loss increased for women from the age of 75 years and over. Dual sensory loss was higher for older women and men who were living alone, with government benefits as their main income source or were divorced, separated or widowed. Dual sensory loss had significant impacts on poor general health, perceived inadequate social activities and community service use for women and men and on depression for women only. Early identification of dual sensory loss is essential to minimize its effects, ensuring continued well-being for this population.

## 1. Introduction

As adults age, they often experience a number of health conditions, including deterioration in vision and hearing. According to the World Health Organization (WHO) [1], there are 253 million people worldwide with vision impairment and 36 million people classified as legally blind. Of the global population with vision impairment, eighty one percent are aged 50 years and over with this percentage increasing as the older adult population increases worldwide. Specifically, the prevalence of low vision amongst older adults (based on test measures) is 4.51% for 60–69-year-olds, 11.41% for 70–79-year-olds, 28.75% for 80–89-year-olds and 39.49% for those 90 years and older [2]. There is also a high prevalence of people worldwide with disabling hearing loss (360 million people or 5.3% of the world’s population) [3]. Hearing loss (as measured by self-report) is exacerbated as people grow older [4]) and approximately one-third of people with hearing loss are aged 65 years and over [5].

Dual sensory loss (DSL) is the combined loss of vision and hearing associated with the aging process [6]. DSL is also known as deafblindness and is a more disabling condition that just the combined effect of vision loss and hearing loss, since those with DSL are a heterogenous group consisting of people with various levels of vision and hearing loss, possible additional disabilities and varying medical aetiologies [7]. DSL is a prevalent disability in older adults. Whilst the absolute number of older adults with DSL is unknown, estimates vary from 3% for those aged 60–69 years to 21.9% for those aged 80 years and over [8]. In an Australian study of older adults, it was reported that the prevalence of DSL increases steadily with age up to 26.8% for those adults aged 80 years and over [9]. Results of a Canadian Longitudinal Study on Aging using audiometry and visual acuity measures, suggested that in 2016, 1.5 million males aged 45–85 years had hearing loss, 1.8 million had vision loss and 570,000 had DSL. Prevalence increased from 8.7% to 16.9% between 2011 and 2016 [10]. Similar results have been obtained in other countries (for example, China) with the prevalence of DSL increasing with age [11].

Eye conditions that are prevalent in older adults are macular degeneration, diabetic retinopathy, cataract, glaucoma and retinitis pigmentosa, with macular degeneration as the leading cause in high-income countries [12]. Women have higher prevalence of moderate and severe vision impairment and blindness than men. Women are also more likely to report more functional difficulties than men as a result of visual impairment [13]. Common causes of hearing loss in older adults include presbycusis, impacted cerumen, chronic otitis media with effusion, noise-induced hearing loss and genetic factors [14]. According to Dammeyer, Rubella Syndrome and Down’s Syndrome are the largest causes of congenital deafblindness amongst adults aged 18 years and over [15]. Cruikshanks et al. [16] investigated the 10 year cumulative incidence of hearing impairment and its associations with education, occupation and noise exposure history. Results of this study suggested that although the risk of hearing impairment was high for both men and women, women experienced a slightly later onset. Hearing sensitivity in men and women is associated with different factors. Poorer hearing sensitivity was associated with high triglyceride levels, high resting heart rate and history of smoking for men. For women, poor hearing sensitivity was associated with high body mass index, high resting heart rate, fast pulse wave velocity, and low Ankle–Arm Index suggestive of peripheral artery disease, putting women at risk of experiencing arterial disease such as stroke [17]. Faster hearing decline is associated with loneliness and being a widow(er) [18], which is a traumatic experience that occurs more frequently in older women than men [18].

With the ageing of populations, the prevalence rate of DSL is set to rise, especially since older adults (those aged 65 years and over) represent the fastest growing segment of the world’s population. In 2010, the older population was 524 million and the World Health Organization [19] has estimated that the older adult population will increase to nearly 1.5 billion by 2050. According to the Australian Government Productivity Commission [20], by 2060, one in six Australians will be aged 75 years or more. By 2050, the number of older adults aged 85 years and over will quadruple from 0.4 million to 1.8 million [21]. There will also be a 25% rise in the number of centenarians living in Australia and older women outnumber older men, since they live longer. This finding is similar to other developed countries, for example, in the U.S. [22]. In 2017, whilst approximately half of all people aged 65–74 years (51%) and 75–84 years (54%) were women, in the older age group (85 years and over), 63% of people were women [23]. Of this population group, many older women are widowed and living alone [24]. Furthermore, the Australian Bureau of Statistics (ABS) reported that over the past two decades, the number of people aged 100 years and over increased by 271%—of which, there were almost four times as many women than men [25]. Sensory devices are often used to improve the sensory capacity. In Australia, eye check and glasses are often used by older Australians with support from Medicare insurance. However, hearing tests and hearing aids are not as well supported by Medicare and are thus less frequently used by older Australians than glasses. In addition, visual aids are not always helpful for people with severe vision loss, and many older adults with hearing loss or DSL do not successfully use amplification or other assistive listening devices successfully to improve their hearing [26].

DSL is a debilitating condition that interferes with communication [27,28], particularly since non-verbal cues such as lipreading are not visible. DSL also inhibits independence, limits one’s activities, restricts participation in society and influences well-being [29,30,31,32]. In a systematic review conducted by Heine and Browning [33], it was found that a range of comorbidities (such as depression and decreased independence) are associated with DSL in older adults. Specifically, a study by Heine, Gong and Browning [11] conducted in China revealed that DSL (as measured by self-report) was significantly and positively associated with advancing age and difficulty in daily living activities. Based on a sample of 21,241 participants aged 45 to 89 years who participated in the Canadian Longitudinal Study on Aging, it was concluded that DSL was independently associated with reduced social network diversity and reduced social participation [34]. These results have been confirmed by Viljanen and colleagues who conducted a cross-sectional study in eleven European countries to evaluate whether sensory difficulties were associated with social inactivity in older adults. Results of this study suggested that sensory difficulties were associated with social inactivity although social inactivity was attenuated by health and socioeconomic indicators [35]. Outcomes of this study also suggested that people with DSL experienced high levels of depression and less life satisfaction [11]. Examining vision loss, hearing loss and DSL separately, Tseng and colleagues conducted a systematic review of the literature and found that increased severity of sensory loss resulted in lower quality of life. Quality of life was also worse for those with DSL as compared to people with vision or hearing loss individually [36]. Older women’s increased longevity [25] possibly contributes to their vulnerability for developing DSL. Older women are not only affected by DSL but also by a number of health, social and economic changes as they age [37]. Older women are more socially and economically vulnerable than older men and more likely to be widowed, divorced, or separated, to depend on government benefits, to be caregivers, or to live alone. Although the majority of older women take measures to prepare for their future care needs, many miss key steps recommended for their future potential well-being [38,39]. Despite coping with difficult circumstances, illness or compromised health (such as being recently widowed, disabled or physically frail), women can remain active and satisfy their needs and aspirations more easily than men [39,40]. Women are, however, potentially disproportionally affected by DSL which may limit their ability to maintain relationships with others and interact in society.

In Australia, 73% of adults aged 65 years and over rate their health as good or better, with the majority living in their own homes with good access to health services [23,25]. However, aging populations are often associated with increases in disability and burden of disease [23]. Managing illness and disability in old age is an important contributor to quality of life and well-being. The aims of this study were to investigate the health inequalities of DSL amongst older women (the most disadvantaged group) by comparing older women to older men. Specifically, this study aimed to:

(1) Describe the prevalence and trajectories of DSL and unmet needs for sensory aids over time and its associated demographic factors and socioeconomic status, in a representative sample of older women (in comparison to men);
(2) Evaluate the associations between DSL and the following variables in women (in comparison to men): living conditions, physical health, mental health, participation in social activities and use of community services at baseline;
(3) Estimate the baseline predictors (including DSL) for ageing in place for both women and men during the whole survey period.

We hypothesized that: (1) DSL will increase with age and its prevalence and related unmet needs will be influenced by socioeconomic status; (2) older women are more likely to live alone and have DSL and other health conditions; and (3) DSL will have a significant influence on physical, mental and social health but affect women differently than men.

## 2. Materials and Methods

### 2.1. Data

The presented analyses utilize data obtained from the Melbourne Longitudinal Studies on Healthy Ageing (MELSHA) Program, which is a 16 year longitudinal study focusing on the physical and mental health of older individuals [41]. The state electoral roll was used to develop a clustered sample of 1422 potentially eligible participants (people aged 65 years and over, who speak English and reside in private dwellings in Metropolitan Melbourne, Australia). The survey did not include people who were living in non-private accommodation, those who could not speak basic English, or those who could not be interviewed for health reason. Excluding these ‘out of scope’ categories, the response rate for the initial interview was 70%, yielding a base sample of 1000 people aged 65 years and over living in Melbourne private dwellings in year 1994 representative of older people in Melbourne at the time.

Over the 16 year period of the study, there were in total 9 waves of data collection, and respondents in the baseline survey were followed up biannually by telephone interviews and by mail in the intervening years. Face-to-face interviews were conducted at baseline and in Waves 7, 8 and 9. Where respondents could not be contacted directly, the tracing procedures relied primarily on next of kin or other individuals volunteered by respondents as key contacts at the time of the baseline interviews. The death data in 2008 and residential care use data in 2006 were linked by resident with the MELSHA longitudinal survey data from 1994 to 2010 [42]. A full description of the cohort profile can be found in Browning and Kendig [41].

The 1994 baseline survey recorded detailed information about respondent’s demographic and socioeconomic characteristics, housing, health status, health behaviours and functioning, medical conditions, care needs and service use. As detailed in Browning and Kendig [41], follow-up survey rounds collected information on a summary set of priority outcome variables. Among the older people who had died during the survey period, most were found to have either died at home or in hospital (deaths after entry to residential care were more difficult to identify). A minority of those who were identified as having left their baseline homes were found to have entered residential care, generally at an advanced age [42]. Entry to residential care or hospital and death were found to have increased rapidly with age.

The MELSHA survey was designed as a longitudinal survey to follow up the changing health, health service use and well-being among older Australians over 16 years (9 waves in total from 1994 to 2010) when their age increased (See Table 1). The participants in the baseline year 1994 were representative of older people aged 65 years and over who spoke English and lived in private dwellings in Metropolitan Melbourne. However, after the baseline year (1994), due to death, moving to residential care or other health issues, there was increasing sample attrition. However, no weight was used to adjust the influence of sample attrition on data representativeness.

After careful consideration of sample attrition and representativeness, the analyses in the current study only used data from 1994 to 2004 (the first 6 waves (10 years) with a sample size from 1000 in 1994 to 326 in 2004. Since the sample size was already very low in 2006 (*n* = 104), the inclusion of the last 3 waves would decrease the precision of our estimations.

The La Trobe University and Monash University Human Ethics Committees approved the study, and signed informed consent was obtained from all participants and participant anonymity was preserved. This ethics approval conforms to the provisions of the Declaration of Helsinki (as revised in Seoul 2008).

### 2.2. Measures

Demographic data collected included age, gender, marital status, living conditions (living alone, housing tenure type) and main income source. Although the focus of this paper is on older women, the results of the older men in this sample have been presented, for comparative purposes.

#### 2.2.1. Measuring Sensory Loss (SL) and Unmet Needs

In the current literature, vision loss and hearing loss have been defined either by passing specific thresholds in medical examinations/testing, for example, Kuang et al. [43] or subjectively using self-reported poor/fair vision and/or hearing capacities, for example, Yu et al. [44]. By following the definition of sensory loss used in previous studies [11,29], in this study, we only used questions relating to self-reported poor/fair vision and hearing to define sensory loss. The two questions relating to self-reported vision capacity included: (1) Do you currently use glasses or contact lenses? and (2) (With your glasses) is your eyesight (excellent/good/fair/poor/functionally or legally blind/totally blind)? The two questions relating to self-reported hearing capacity included: (1) Do you currently use a hearing aid? and (2) Is your hearing (with your hearing aid) (excellent/good/fair/poor)? Poor vision (or vision loss) was defined as self- reported poor/fair eyesight (with glasses). The unmet need for glasses was defined as having poor/fair vision while not currently using glasses or contact lenses. Poor hearing (or hearing loss) was defined as self- reported poor/fair hearing (using a hearing aid). The unmet need for hearing aids was defined as having poor/fair hearing while not using a hearing device. Dual sensory loss (DSL) was defined as self-reported vision loss plus hearing loss.

The longitudinal nature of MELSHA data also allows for the examination of the changes of hearing, vision and DSL over 2 years, 6 years and 10 years from 1994 to 2004. Data after 2004 were not included in this study, since the sample size was extremely small mainly due to mortality (See Table 1).

#### 2.2.2. Other Measures

Self-rated general health was measured according to respondent’s answers when they were asked to rate their own health compared to people of the same age. This variable was regrouped into two categories based on data distribution: (1) excellent/very good/good; (2) fair/poor. Mental health (as measured by the presence or absence of depression) was measured using a cut-off score of 5 or more on the Psychogeriatric Assessment Scales (PAS) [45] (score ranges were 0–12). Perceived current social activity level was used, where 1 = not enough and 0 = about right or too much to identify social health, as it represents both need and practice. Use of community services was categorised as ‘yes’ if a respondent answered ‘yes’ to either of the two questions: “Do you use any organized community services such as the home and community care program?”; “Who helps you with household duties or personal care which you cannot do on your own? Organized community services (e.g., home and community care program)?”.

“Ageing in place” in this study is defined as a longitudinal outcome during the ten years study period (from 1994 to 2004) and was used instead of a current outcome for any of the survey years. “Ageing in place” refers to women (or men) who had remained in their 1994 residence until the survey year of 2004, or within two years before their death. Those women (or men) who had moved or entered residential care either before the last wave of the survey or two years before their death were classified as “not aged in place” [42]. Some people in the sample could not be defined due to attrition (being “alive but not in the survey”) after 1994. During the 10 years from 1994 to 2004, 176 women were defined as “aged in place”, 120 women were defined as “not aged in place” and 237 women could not be defined. From 1994 to 2004, there were 249 men defined as “aged in place”, 76 men defined as “not aged in place” and 142 men could not be defined due to attrition.

### 2.3. Statistical Analyses

The longitudinal nature of MELSHA data has been used to: (1) investigate the prevalence and persistence of DSL over time in the baseline year, and after 2, 6 and 10 years based on data in 1994, 1996, 2000 and 2004 and (2) to estimate the baseline predictors of ageing in place which were defined based on the 10 years survey data from 1994 to 2004. Unlike other health conditions which were more likely to be chronic and had long term impact, and although the prevalence of dual sensory loss (DSL) increased as a result of ageing, DSL could be improved greatly by using glasses and/or hearing aids. Consequently, this study aimed to investigate the health inequalities in terms of the prevalence of DSL, unmet needs for glasses/hearing aids and their current impacts on service use and well-being. Long-term impacts of DSL on health service use and well-being were not felt to be meaningful, as there were very few instances of persistent DSL over time. Consequently, it was decided to use the baseline full sample data to investigate the current impact of DSL on service use and well-being, as well as the baseline predictors of ageing in place during 10 years, while conducting the robustness check of regression results using 1994 data including both women and men in one model, as well as using 2004 data for women and men separately in two models.

Although the pooled data of all waves had been initially investigated, there were several reasons as to why the pooled data of all waves was not reported in our final analysis—reasons include concern about autocorrelation and dependence of the residuals generated by using the pooled data from longitudinal survey with repeated individuals and secondly, concern regarding the lack of weights to adjust for the impact of sample attrition. In addition, unmet need for glasses and hearing aids cannot be defined consistently in years 1998 and 2002. The confidence level of 90% (or an Alpha level of 10% relates to a Type 1 error) was used to test the significance of our estimates in this study due to the relatively small sample size of the MELSHA survey.

## 3. Results

### 3.1. Descriptive Analysis

Overall, at baseline, there were 1000 people in the total sample, with 533 women and 467 men. See Table 2.

At baseline (in 1994), there were 326 women aged 65–74 years and 207 women aged 75 years and over, compared to 316 men aged 65–74 years and 151 men aged 75 years and over. Amongst the 533 women, 38.7% were currently living with a partner, whilst 56.5% were widowed, divorced or separated, and 4.9% never married. In 2004, the sample size decreased for both men and women to 166 women and 148 men, all aged 75 years and over. Consistent with the trends in 1994, in 2004, 32.3% of women were living with a partner, whilst 64.0% were widowed, divorced or separated and 3.7% never married. As is evident from Table 2, women were more likely to have no partner, live alone, be dependent on government income or be public renters compared to men in both 1994 and 2004.

Examining vision loss, hearing loss, and DSL and good vision and hearing separately, a proportion of women consistently reported poor vision in 1994 (*n* = 94; 17.8%) and in 2004 (*n* = 18; 10.9%). In 1994, 80 women (15.2%) reported poor hearing, and this proportion increased to 20.6% (*n* = 34) in 2004 when age increased. The number of men reporting poor vision (*n* = 51; 11.0%) or poor hearing (*n* = 106; 22.8%) in 1994 proportionately decreased in 2004 for poor vision (*n* = 13; 8.8%) and increased for poor hearing (*n* = 46; 31.3%). In both 1994 and 2004, women had a higher proportion of poor vision, while men had a higher proportion of poor hearing. The proportion of DSL increased from 1994 to 2004 (11.2% to 18.8% for women and from 11.0% to 12.9% for men) when age increased. The rate of unmet needs for hearing aids in 1994 was higher for men than women in both years of 1994 and 2004 (25.0% for men compared to 20.8% for women in 1994; 22.5% for men compared to 20.6% for women in 2004). Unmet needs for glasses were low (less than 1%) and hence not reported further. It was also evident that the proportion of older women with good vision and hearing decreased from 55.9% (*n* = 295) in 1994 (total sample of women = 533) to 49.7% (*n* = 82) in 2004 (total sample *n* = 166).

Table 3 presents the sensory capacity and the use of glasses and hearing aids in years 1994, 2000 and 2004. From Table 3, it is evident that the prevalence rate of DSL in the cohort increased over the ten years study period, with little and no significant differences between the genders. Women were more likely to report poor vision, while men were more likely to have poor hearing. Though the proportion of poor hearing was only slightly lower than that of poor vision, the majority (more than 90%) of older Australian women and men had glasses since earlier old ages, while fewer older women and men (11–37%) used hearing aids. The proportion of wearing glasses and poor vision were relatively constant over time when aged increased, while the proportion of poor hearing and using hearing aids increased by age. The unmet need for glasses was lower than 2%, while the unmet need for hearing aids was around 20–25%.

Unlike other chronic diseases, DSL might be improved by using glasses and/or hearing aids. Therefore, whether the sensory loss was for a short term or was persistent for a longer time was examined by investigating the transitions to DSL as it occurred in 2 years, 6 years, and 10 years post-baseline (See Table 4).

As is apparent from Table 4, persistent DSL for women was higher in the 2 years post baseline, whilst it was very similar at about 5 and 10 years post baseline (5.4% in 2 years, compared to 3.2% in 6 years and 3.1% in 10 years). In comparison, for men, persistent DSL decreased from a short-term to a long-term condition (4.9% at 2 years post-baseline compared to 4.0% at 6 years and 3.4% at 10 years post-baseline). Women had a relatively higher proportion of persistent vision loss (14.6%, 12.2% and 9.7% for women compared to 11.0%, 10.4% and 8.8% for men in 2 years, 6 years and 10 years post-baseline respectively), while men had a relatively higher proportion of persistent hearing loss (27.4%, 25.2% and 21.1% for men when compared to 18.3%, 15.8% and 14.7% for women in 2 years, 6 years and 10 years post-baseline respectively). For both men and women, the proportion of persistent hearing loss was higher than that of persistent vision loss at any of the three time points.

In regards to the demographic factors associated with DSL, it was apparent that in 1994 (see the first part of Table 5), the prevalence of DSL for women was significantly higher amongst those aged 75 years and over, previously with a partner (widowed, separated or divorced), living alone or those with public income as their main source of income. In 1994, the prevalence of DSL was similar among women and men (11.2% compared to 11.0%), while it became much higher in women than for men after 10 years, in 2004 (18.8% when compared to 12.9%). For men, the prevalence of DSL in 1994 was significantly higher amongst those previously living with a partner, living alone, or for those with public income as their main source of income.

In comparison, in 2004 (see the second part of Table 5), the prevalence of DSL increased for women after 10 years (from 11.2 % to 18.8%) and was still significantly higher amongst those women without a partner (widowed, separated or divorced), living alone or with public income as their main source of income. The prevalence of DSL for men had increased from 11.0% in 1994 to 12.9% in 2004 and was still higher amongst those men without a partner (widowed, separated or divorced) (16.2% in 1994 and 17.9% in 2004), but became lower for those living alone (15.7% in 1994 and 12.5% in 2004). The gap in the proportion of those with DSL by major income source from government benefits were larger in both years (1994 and 2004) for women (14.2% compared to 4.0% in 1994 and 22.0% compared to 12.5% in 2004), whilst this gap decreased for men (12.7% compared to 7.8% in 1994 and 13.9% compared to 10.3% in 2004).

The number of medical conditions for women and men with or without DSL is shown in Table 6. It is apparent from Table 6 that in 1994, older women with DSL or poor vision had the highest number of medical conditions (5.1 and 5.2 respectively) compared to women with good sensory acuity or “hearing loss only”. In 2004 with advancing age, older women with DSL or poor hearing had the highest number of medical conditions (4.6 and 5.2 respectively) compared to those with good sensory acuity or “poor vision only”. In contrast, the variation in the number of medical conditions related to poor vision or hearing were much smaller for men compared to women.

Due to the small sample size remaining in 2004, we used the 1994 data to examine the univariate and multivariate associations between DSL and health and social outcomes. Table 7 reports the cross-tabulations of women and men with a range of sensory acuities and the self-reported outcomes including living conditions, health, depression, social activities, community services and ageing in place. From the top part of Table 7, it is apparent that amongst older women with DSL: (1) 69.5% were living alone (which is a much higher percentage than women with no sensory loss (46.8%) or one sensory loss only (46–48%); (2) 34.5% reported poor/fair general health, followed by 29.8% for those with vision loss, compared to women with no sensory loss (13.3%) or those with hearing loss (10.1%); (3) 35.6% were depressed, followed by 26.7% of women with vision loss, 22.5% of women with hearing loss, compared to those with no sensory loss (12.9%); (4) 24.1% perceived inadequate social activity, compared to those with hearing loss (29.9%), with vision loss (19.4%) or no sensory loss (16.3%); (5) 23.7% used community services, compared to those with hearing loss (25.0%), vision loss (18.2%) and those with no sensory loss (10.8%) and (6) 47.2% did not have ageing in place, closely followed by those with no sensory loss (41.3%), and those with hearing loss (40.0%) compared to those with vision loss (33.3%).

There was less variation in DSL prevalence by individual characteristics in men compared to women (see the bottom part of Table 7). For older men with DSL, the following is apparent: (1) they were the most likely to report poor/fair health (29.4%), perceive inadequate social activities (26.0%), use community services (15.2%) and live alone (21.6%); (2) older men with poor hearing, were the most likely to report being depressed (16.0%) and (3) older men with poor vision, were the most likely not to have ageing in place (31.6%).

### 3.2. Regression Results

Multivariate regression analysis based on 1994 outcomes or predictors is shown in Table 8 and full regression results in Table S3 for women and Table 9 and Table S4 for men. As is apparent from Table 8, after controlling for other variables, for women: (1) having DSL had a significant impact on self-reported general health, depression, inadequate social activities and community service use; (2) “poor vision only” had a significant impact on self-reported general health and depression; (3) “poor hearing only” had a significant impact on depression, inadequate social activities and community service use.

There are also some common and some different influence of the type of sensory loss experienced by women including: (1) DSL, poor vision only or poor hearing only all had no significant influence on ageing in place but all had a significant influence on depression; (2) poor vision had a significant influence on self-reported health, while poor hearing had a significant influence on community service use and DSL on both; and (3) poor hearing and DSL, but not poor vision, had a significant influence on perceived inadequate social activities indicating that social participation and independence can be improved for those with poor hearing and DSL.

Further findings from the full regression model (Table S3) show that: (1) those with private income sources were less likely to have DSL, while those living alone were more likely to have DSL; (2) The unmet need for hearing aids was associated with living alone but was not significantly related to other socioeconomic status (SES). This confirms that women living alone were the most disadvantaged in terms of having DSL and the unmet needs for hearing aids.

For comparative purposes, we have also reported the multiple regression results for men in Table 9 (and Table S4). The comparison of the estimated coefficients of sensory loss for women to men (Table 8 to Table 9) shows that there are some commonalities and differences in the findings for men and women and are as follows: (1) DSL had a significant influence on self-reported general health, community service use and inadequate social activities for both women and men, whilst DSL had a significant impact on depression for women only; (2) “Poor vision only” had a significant impact on self-reported general health and depression amongst women only, and on inadequate social activities amongst men only; (3) “Poor hearing only” had a significant impact on depression and inadequate social activities for both women and men, on community service use in women only, and on self-reported health in men only; and (4) there was no significant influence of DSL, poor vision or poor hearing on ageing in place for both women and men.

The further comparison of full regression results of women to men (Table S3 to Table S4) indicates that: (1) living alone was positively associated with both DSL and the unmet need for hearing aids among women; (2) being a private renter was positively associated with the unmet need for hearing aids among men; and (3) private income was negatively associated with DSL for both men and women.

### 3.3. Robustness Check

Women and men generally have different social and health outcomes. Table 10 (and Table S5) reports the multiple regression results for persons (including both women and men in one model) with gender controlled as a predictor. The significant influence of DSL, poor vision, poor hearing on social and health outcomes are found to be similar and robust as shown in Table 8.

Since there was increasing sample attrition (attributed to death or other health issues) after the baseline year (1994), the robustness of the regression results (evident in Table 8 for women in 1994 and Table 9 for men in 1994) was checked using 2004 data and can be viewed in Table 11 for women in 2004 and Table 12 for men in 2004. As is evident from Table 11, the significant influence of DSL on self-reported general health, depression, perceived social activities and community service use; and the influence of poor hearing on perceived social activities and community service use amongst women found in 1994 are robust when using the 2004 data although the sample size in 2004 is much smaller than in 1994.

For men, the influence of poor vision on perceived inadequate social activities and the influence of DSL on poor/fair self-reported general health are still significant in 2004 as in 1994, while the significant influence of poor vision on self-reported poor health and DSL on depression are new in 2004. This indicates that the influence of DSL across ages is consistent for women, while it varies for men (See Table 12).

## 4. Discussion

Overall, the results of this study suggested that DSL is of increasing concern when age increased for older Australians aged 65 and over, especially for Australian women who were living alone as they age. On average, older women were found to have a lower socioeconomic status (e.g., a higher proportion of living in public housing and depending on government benefits), were more likely to live alone with DSL and live longer than men. The combination of DSL with other health conditions could represent an added burden for older women.

Furthermore, more than half of the women in this study were either divorced, separated or widowed and living alone. These circumstances, combined with DSL, present women with the challenge of establishing other relationships or taking up opportunities that will differ to those women in spousal relationships. Living alone following a change in marital status or losing their partners highlights the centrality of continued connections with family, friends and community. Whilst many older women are determined to continue to engage with the world around them regardless of age or health circumstances, barriers may emerge. Engaging with other people as a single older woman and coping with DSL may prove challenging particularly when facing decreased independence and the possible threat of ill health as they age.

Good health is essential to carrying out day-to-day activities. In this study, self-rated health, depression, decreased social activity and use of community services was significantly poorer in women with DSL and confirms results of previous studies [30,46]. The impacts of declining physical and social health and DSL are numerous, affecting mobility, social connectedness and one’s sense of well-being. These impacts are potentially further compounded for older women living alone, as is the case for the majority of older women in this study. Most aged care policy approaches aim to support people to live in their homes for as long as possible [42,47,48]. Our findings confirm that “poor vision only”, “poor hearing only” or DSL, had no significant impact on ageing in place among older women (as well as older men) after controlling for other variables, reflecting that older women (and older men) could still age in place even if they had sensory loss. Additional support is thus required for these older women (and older men) with DSL if living at home for as long as possible is to be encouraged and upheld.

The relationship between mental health and DSL has been previously explored in a systematic review [31]. A number of studies have found a significant relationship between DSL and depressive symptoms [49,50,51]. The relationship between DSL and depression was confirmed in this study in that older women with DSL or poor vision or hearing had more occurrences of depression compared to older women with no sensory loss. In general, older women had a higher proportion of depression (19.3%) when compared to older men (10.5%); and having DSL, poor vision or poor hearing increased the likelihood of depression dramatically for women (from 12.9% to 35.6%, 26.7% and 22.5%, respectively), whilst having poor hearing increased depression for men greatly (from 7.4% to 13.7%). This might reflect women’s role as a care giver whilst men are care receivers in later life. There have been no studies to date that have evaluated gender differences and mental health in older people with DSL even though in the general older adult population, more women than men experience depression [52].

All social and health outcomes explored in this study were generally poorer in those women with DSL, when compared to women with good hearing and vision. This suggests that older women with DSL experienced a range of health and psychosocial issues as they aged. In particular, DSL in older women was significantly correlated with poorer general health, higher occurrence of depression, less social activity and use of community services. Social activities and use of community services were significantly influenced in women with DSL or poor hearing. This could possibly be attributed to the fact that women were more likely to experience widowhood hence live alone [53].

A number of recommendations for older women are thus indicated, including the provision of programs for older women with DSL that would greatly benefit their mental health and well-being. Early detection of DSL and timely rehabilitation is important [54]. Professionals working with older women should also be aware of the combined effects of sensory loss on individuals [55] and the high prevalence of DSL in this population and its impacts. Since DSL is a multidisciplinary disorder often involving assessment by optometrists, opthalmologists, audiologists and otolaryngologists as well as other medical and allied health professionals, interdisciplinary collaboration in identification, assessment and management of DSL and its associations is required to ameliorate the impact of DSL in older women. Education for professionals working with older adults with DSL is warranted to ensure collaborative care is adequately provided [56].

Recently, specific interventions for people with DSL have been discussed in the literature. For example, Roets-Merken et al. [57] and Roets-Merken et al. [58] reported a protocol for a trial to examine the effectiveness of a self-management program for older people with DSL in aged care settings. Such approaches hold promise, especially since self-management approaches have been successfully implemented in older people with other chronic conditions [59]. The few studies that are available examining the effectiveness of programs for people with DSL are often poorly designed, have taken a narrow view (for example, sensory aid usage) as opposed to interventions investigating treatments leading to broader health and well-being outcomes and only few studies have delivered good rehabilitation outcomes. A review of rehabilitation interventions to improve emotional and functional status in people with sensory loss found little evidence for their effectiveness [58].

The limitations of the current study included that the survey used was representative of the older (aged 65 years and over) population in 1994 through to 2004 and in Melbourne only, not the whole of Australia. A more recent and updated survey on the ageing population covering all Australians such as the Health and Retirement Survey (HRS) in the United States (U.S.) is urgently required. In addition, participants may have responded according to their perceived social desirability of their responses, and some participants may have minimized the extent of their sensory loss for this reason. Longitudinal studies are subject to selection bias whereby participants died or were lost-to-follow up and this affects the generalizability of the results. Finally, the estimations of DSL transitions over time need to be treated cautiously due to the small sample size. Nonetheless, we estimate baseline predictors and conditional and unconditional associations between DSL and other social and health outcomes but advise caution in any attempt to interpret our results in terms of causality.

## 5. Conclusions

DSL is a significant health issue for older people that impacts on general health and well-being and the ability to engage fully in life activities. For women who live alone, this is particularly problematic. The increase in the prevalence of DSL and unmet need for hearing aids as populations age requires a concerted effort on the part of researchers and service providers to ensure that effective and efficient programs and interventions are tested and made available to older women and men with DSL.

## Figures and Tables

**Table 1:** Participants across waves 1994–2010.

Sample Size	1994	1996	1998	2000	2002	2004	2006	2008	2010
Total in survey	1000	789	646	589	438	326	104	201	132
(1) In 1994 residence	1000	759	613	507	NA	300	NA	181	127
(2) Not in 1994 residence		22	27	21	NA	17	NA	17	5
(3) Unknown residence		8	6	8	NA	9	NA	3	0
(4) No reply for this question				53					
In residential care and still alive		18	31	39	39	34	35	34	34
In residential care and died		33	51	61	81	107	116	117	117
Not in residential care and died		55	101	146	217	271	310	331	331
Alive but not in survey		105	171	165	225	262	435	317	386
Total		1000	1000	1000	1000	1000	1000	1000	1000

Data source: Melbourne Longitudinal Studies on Healthy Ageing (MELSHA) survey data 1994 to 2010. Only data from 1994 to 2004 was used in the current study.

**Table 2:** Individual characteristics, prevalence of dual sensory loss (DSL) and unmet needs by gender in 1994 and 2004.

Individual Characteristics		Year 1994				Year 2004			
		Women		Men		Women		Men	
Variables	Values	*n*	%	*n*	%	*n*	%	*n*	%
All	All	533	100	467	100	166	100	148	100
Age group	65–74	326	61.2	316	67.7				
	75+	207	38.8	151	32.3	166	100	148	100
Marital status	Currently living with partner	206	38.7	372	79.7	53	32.3	110	76.4
	Previously with a partner	301	56.5	75	16.1	105	64.0	28	19.4
	Never married	26	4.9	20	4.3	6	3.7	6	4.2
Living alone	No	269	50.5	396	84.8	68	41.5	114	78.1
	Yes	264	49.5	71	15.2	96	58.5	32	21.9
Tenure	Own house	441	83.7	412	88.2	146	89.0	134	91.8
	Paying off house	16	3.0	21	4.5	1	0.6	2	1.4
	Public renter	25	4.7	9	1.9	5	3.1	1	0.7
	Private renter	19	3.6	15	3.2	4	2.4	1	0.7
	Other	26	4.9	10	2.1	8	4.9	8	5.5
Income sources	Government benefits	368	70.6	293	63.7	110	69.6	79	57.7
	Private income	153	29.4	167	36.3	48	30.4	58	42.3
Dual Sensory Loss (DSL)	Good vision and hearing	295	55.9	257	55.3	82	49.7	69	46.9
	Poor vision only	94	17.8	51	11.0	18	10.9	13	8.8
	Poor hearing only	80	15.2	106	22.8	34	20.6	46	31.3
	Dual sensory loss	59	11.2	51	11.0	31	18.8	19	12.9
Unmet needs for hearing aids	No unmet needs	418	79.2	349	75.1	131	79.4	114	77.6
	With unmet needs	110	20.8	116	25.0	34	20.6	33	22.5

Data source: MELSHA 1994–2004. Note: The numbers in the cells with a sample size smaller than 20 should be interpreted with caution. Due to some small but different missing information for each of the variables, the sum of the numbers at the top by age group, is slightly different from the addition of some of the other variables. Table S1 presents the prevalence rate of DSL among older Australians aged 65 and over for women compared to men in all waves from 1994 to 2004 by age group.

**Table 3:** Sensory capacity and the use of glasses and hearing aids in 1994, 2000 and 2004.

Sensory Capacity and Device Use	Women			Men		
	1994	2000	2004	1994	2000	2004
1.1 Wearing glasses	96.3%	93.4%	95.2%	96.2%	93.2%	95.2%
1.2 Poor vision (with glasses if there are)	28.9%	26.0%	29.5%	22.3%	23.6%	21.8%
1.3 Unmet needs for glasses	1.0%	2.4%	1.8%	1.1%	1.2%	0.7%
2.1 Using hearing aids	11.6%	15.6%	28.5%	18.0%	31.2%	37.0%
2.2 Poor hearing (with aids if there are)	26.3%	32.3%	39.4%	33.8%	46.0%	44.2%
2.3 Unmet need for hearing aids	20.8%	23.3%	20.6%	25.0%	24.0%	22.5%
3. DSL	11.2%	14.9%	18.8%	11.0%	16.8%	12.9%

Data source: MELSHA 1994–2004.

**Table 4:** Persistent DSL, vision loss and hearing loss in 2 years, 6 years and 10 years post baseline.

Persistence of DSL	Women		Men	
	*n*	%	*n*	%
DSL in both 1994 and 1996	23	5.4%	18	4.9%
DSL in both 1994 and 2000	9	3.2%	10	4.0%
DSL in both 1994 and 2004	5	3.1%	5	3.4%
Poor hearing in both 1994 and 1996	78	18.3%	100	27.4%
Poor hearing in both 1994 and 2000	45	15.8%	63	25.2%
Poor hearing in both 1994 and 2004	24	14.7%	31	21.1%
Poor vision in both 1994 and 1996	62	14.6%	40	11.0%
Poor vision in both 1994 and 2000	35	12.2%	26	10.4%
Poor vision in both 1994 and 2004	16	9.7%	13	8.8%

Data source: MELSHA 1994–2004. Note: The numbers in the cells with a sample size smaller than 20 should be interpreted with caution. For more details, see Table S2.

**Table 5:** DSL by individual characteristics for women and men in 1994 and 2004.

Individual Characteristics		With DSL 1994				With DSL 2004			
		Women		Men		Women		Men	
Variables	Values	*n*	%	*n*	%	*n*	%	*n*	%
All	All	59	11.2	51	11.0	31	18.8	19	12.9
Age group	65–74	32	9.9	36	11.4				
	75+	27	13.3	15	10.1	31	18.8	19	12.9
Marital status	Currently living with partner	14	6.8	37	10.0	6	11.3	12	11.0
	Previously with a partner	44	14.8	12	16.2	24	23.1	5	17.9
	Never married	1	4.0	2	10.0	1	16.2	1	16.7
Living alone	No	18	6.7	40	10.1	8	11.9	15	13.3
	Yes	41	15.7	11	15.7	22	22.9	4	12.5
Tenure	Own house	47	10.7	45	11.0	25	17.2	16	12.0
	Paying off house	1	6.3	5	23.8	0	0.0	0	0.0
	Public renter	3	12.0	0	0.0	0	0.0	0	0.0
	Private renter	2	10.5	0	0.0	1	25.0	0	0.0
	Other	6	23.1	1	10.0	4	50.0	2	25.0
Main income resource	Government benefits	52	14.2	37	12.7	24	22.0	11	13.9
	Private income	6	4.0	13	7.8	6	12.5	6	10.3

Data source: MELSHA 1994–2004. Note: The numbers in the cells with a sample size smaller than 20 should be interpreted with caution. Due to some small but different missing information for each of the variables, the sum of the numbers at the top by age group, is slightly different from the addition of some of the other variables.

**Table 6:** Comorbidity of DSL with other medical conditions in 1994 and 2004.

Comorbidity of DSL with Medical Conditions	Women		Men	
Sensory Status	Number of Medical Conditions 1994 (Mean)	Number of Medical Conditions 2004 (Mean)	Number of Medical Conditions 1994 (Mean)	Number of Medical Conditions 2004 (Mean)
Good vision and hearing	2.6 (296)	3.0 (82)	3.0 (257)	3.9 (69)
Poor vision only	5.2 (94)	3.1 (18)	3.2 (51)	3.6 (13)
Poor hearing only	3.0 (80)	5.2 (34)	4.0 (106)	3.4 (46)
Dual sensory loss	5.1 (59)	4.6 (31)	3.3 (51)	2.9 (19)

Data source: MELSHA survey data; no weights were used. Notes: Wave 1 (in 1994): 25 conditions asked. Wave 6 (in 2004): 17 conditions asked. The numbers in brackets are the sample size. The estimations based on a sample size smaller than 20 should be interpreted with caution.

**Table 7:** DSL and associated social and health outcomes in 1994.

**Women**	**ALL**	**Living Alone (1994)**		**Self-Reported Poor/Fair Health 1994**		**Depressed 1994**		**Inadequate Social Activities 1994**		**Community Service Use 1994**		**Not Aged in Place 1994–2004**	
DSL 1994	*n*	*n*	%	*n*	%	*n*	%	*n*	%	*n*	%		%
Good vision and hearing (no-SL)	295	138	46.8	39	13.3	38	12.9	48	16.3	30	10.8	64	41.3
Poor vision only (VL)	94	44	46.8	28	29.8	25	26.7	18	19.4	16	18.2	19	33.3
Poor hearing only (HL)	80	38	47.5	8	10.1	18	22.5	23	29.9	18	25.0	18	40.0
Dual sensory loss (DSL)	59	41	69.5	20	34.5	21	35.6	14	24.1	14	23.7	17	47.2
All women	528	261	49.4	95	18.1	102	19.3	103	19.7	78	15.7	118	40.3
**Men**	**ALL**	**Living Alone**		**Self-Reported Poor/Fair Health 1994**		**Depressed 1994**		**Inadequate Social Activities 1994**		**Community Service Use 1994**		**Not Ageing in Place 1994–2004**	
DSL 1994	*n*	*n*	%	*n*	%	*n*	%	*n*	%	*n*	%	*n*	%
Good vision and hearing	257	40	15.6	36	14.2	19	7.4	31	12.1	13	5.4	36	21.2
Poor vision only	51	9	17.7	10	20.0	6	11.8	11	21.6	1	2.0	12	31.6
Poor hearing only	106	10	9.4	21	20.0	17	16.0	22	20.8	5	5.3	19	23.5
Dual sensory loss	51	11	21.6	15	29.4	7	13.7	13	26.0	7	15.2	9	26.5
All men	465	70	15.1	82	17.8	49	10.5	77	16.6	26	6.1	76	23.4

Data source: MELSHA 1994–2004. Note: The numbers in the cells with a sample size smaller than 20 should be interpreted with caution.

**Table 8:** Multivariate regression results on DSL prevalence and its associated outcomes in 1994 for women.

Dependent Variables for Women	DSL 1994	Unmet Needs for Hearing Aids 1994	Poor/Fair Health	Depressed 1994	Perceived Inadequate Social Activities 1994	Community Service Use 1994	Aged in Place 1994–2004
Sensory capacity 1994	Estimated coefficient						
Good vision and hearing (Ref. group)							
Poor vision only			1.035 *	0.762 *	0.257	0.353	0.590
Poor hearing only			−0.229	0.580 *	0.799 *	0.957 *	0.132
Dual sensory loss			1.410 *	1.237 *	0.616 *	0.687 *	−0.199
Constant	−2.284	−1.433	−1.754	−2.299	−1.691	−2.849	0.906
R-square	0.079	0.012	0.079	0.0547	0.022	0.1087	0.062
Sample	517	517	514	517	493	488	287

Data source: MELSHA survey data; no weights were used. Note: * indicates significance at the 10% level. Major demographics and socioeconomic status (including age group, gender, marital status, living alone, housing tenure type, main income source) are controlled for in all these regression models. The estimated coefficients from the full models are reported in Table S3. Ref. group = reference group.

**Table 9:** Multivariate regression results on DSL prevalence and its associated outcomes, 1994 men.

Dependent Variables for Men	DSL 1994	Unmet Needs for Hearing Aids 1994	Self-Reported Poor/Fair Health 1994	Depressed 1994	Perceived Inadequate Social Activities 1994	Community Service Use 1994	Aged in Place 1994–2004
Sensory capacity 1994	Estimated coefficient						
Good vision and hearing (Ref. group)							
Poor vision only			0.424	0.572	0.676 *	−1.328	−0.580
Poor hearing only			0.531 *	0.855 *	0.663 *	0.021	−0.289
Dual sensory loss			0.832 *	0.528	1.032 *	1.265 *	−0.317
Constant	−2.021	−1.188	−1.687	−2.828	−2.277	−3.194	2.006
R-square	0.0293	0.0218	0.0616	0.0539	0.056	0.158	0.097
Sample	435	458	453	450	457	394	318

Data source: MELSHA survey data; no weights were used. Note: * indicates significance at the 10% level. Major demographics and socioeconomic status (including age group, gender, marital status, living alone, housing tenure type, main income source) are controlled for in all these regression models. The estimated coefficients from the full models are reported in Table S4. Ref. group = reference group.

**Table 10:** Multivariate regression results on DSL prevalence and its associated outcomes in 1994, all persons.

Dependent Variables for Both Women and Men	DSL 1994	Unmet Needs for Hearing Aids 1994	Poor/Fair Health 1994	Depressed 1994	Perceived Inadequate Social Activities 1994	Community Service Use 1994	Aged in Place 1994–2004
Sensory capacity 1994	Estimated coefficient						
Good vision and hearing (Ref. group)							
Poor vision only			0.771 *	0.694 *	0.392	0.071	0.247
Poor hearing only			0.196	0.702 *	0.700 *	0.679 *	0.006
Dual sensory loss			1.135 *	0.983 *	0.762 *	0.808 *	−0.210
Constant	−1.996	−1.145	−1.71462	−2.80738	−2.059	−3.403	1.611
R-square	0.046	0.011	0.044	0.064	0.030	0.133	0.074
Sample	975	975	967	975	969	911	605

Data source: MELSHA survey data; no weights were used. Note: * indicates significance at the 10% level. Major demographics and socioeconomic status (including age group, gender, marital status, living alone, housing tenure type, main income source) are controlled for in all these regression models. The estimated coefficients from the full models are reported in Table S5. Ref. group = reference group.

**Table 11:** Multivariate regression results on DSL incidence and its influences, 2004 women.

Dependent Variables for Women	DSL 2004	Unmet Needs for Hearing Aids 2004	Poor/Fair Health 2004	Depressed 2004	Perceived Inadequate Social Activities 2004	Community Service Use 2004	Aged in Place 1994–2004
Sensory capacity 2004	Coef.	Coef.	Coef.	Coef.	Coef.	Coef.	Coef.
Good vision and hearing (Ref. group)							
Poor vision only			0.128	−0.311	1.048	0.192	0.000
Poor hearing only			0.037	−0.010	1.788 *	0.845 *	0.239
Dual sensory loss			2.449 *	1.374 *	1.940 *	1.024 *	−2.175
Constant	−2.532	−1.590	−2.194	−1.502	−3.481	−1.576	3.220
R-square	0.0899	0.0236	0.155	0.0608	0.1419	0.099	0.306
Sample	145	145	146	144	150	139	56

Data source: MELSHA survey data; no weights were used. Note: * indicates significance at the 10% level. Major demographics and socioeconomic status (including age group, gender, marital status, living alone, housing tenure type, main income source) are controlled for in all these regression models. The estimated coefficients from the full models are reported in Table S6. Ref. group = reference group.

**Table 12:** Multivariate regression results on DSL incidence and its influences, 2004 men.

Dependent Variables for Men	DSL 2004	Unmet Needs for Hearing Aids 2004	Poor/Fair Health 2004	Depression 2004	Perceived Inadequate Social Activities 2004	Community Service Use 2004	Aged in Place 1994–2004
Sensory capacity 2004	Coef.	Coef.	Coef.	Coef.	Coef.	Coef.	Coef.
Good vision and hearing (Ref. group)							
Poor vision only			1.944 *	1.180	1.909 *	−0.897	−1.052
Poor hearing only			0.529	0.980	0.745	0.275	−0.876
Dual sensory loss			1.371 *	2.889 *	0.717	0.896	0.000
Constant	−1.942	−0.745	−1.907	−3.112	−2.260	−1.217	2.661
R-square	0.0251	0.1091	0.141	0.201	0.102	0.130	0.057
Sample	127	123	124	122	129	121	56

Data source: MELSHA survey data; no weights were used. Note: * indicates significance at the 10% level. Major demographics and socioeconomic status (including age group, gender, marital status, living alone, housing tenure type, main income source) are controlled for in all these regression models. The estimated coefficients from the full models are reported in Table S7. Ref group = reference group.

## Supplementary Materials

The following are available online at https://www.mdpi.com/1660-4601/17/1/263/s1. Table S1: Prevalence of Sensory Loss by age group for women and men in all waves from 1994 to 2004. Table S2: Persistence of DSL, hearing loss and vision loss at baseline plus 2 years, plus 6 years and plus 10 years. Table S3: Multivariate regression results for DSL prevalence and its associated outcomes in 1994 for women. Table S4: Multivariate regression results for DSL prevalence and its associated outcomes in 1994 for men. Table S5: Multivariate regression results for DSL prevalence and its associated outcomes in 1994 for men and women. Table S6: Multivariate regression results for DSL incidence and its influences in 2004 for women. Table S7: Multivariate regression results for DSL incidence and its influences in 2004 for men.
