# Supplementary material for: Older Women in Australia: Facing the Challenges of Dual Sensory Loss
Source: Int J Environ Res Public Health. 2019 Dec 30;17(1):263. doi: 10.3390/ijerph17010263 (PMC6981467; doi:10.3390/ijerph17010263)
Supplement: Supplementary file 1 [file ijerph-17-00263-s001.pdf]

Table S1. Prevalence of Sensory Loss by age group for women and men in all waves from 1994 to 2004.

Age	Sensory loss	Women 1994		Women 1996		Women 1998		Women 2000		Women 2002		Women 2004	
All 65+	Good vision and hearing	295	55.9	244	57.0	198	56.4	163	56.6	99	46.3	82	49.7
All 65+	Poor vision only	94	17.8	53	12.4	37	10.5	32	11.1	35	16.4	18	10.9
All 65+	Poor hearing only	80	15.2	75	17.5	67	19.1	50	17.4	48	22.4	34	20.6
All 65+	Dual sensory loss	59	11.2	56	13.1	49	14.0	43	14.9	32	15.0	31	18.8
Aged 65-74	Good vision and hearing	198	60.9	130	58.0	90	65.7	45	67.2	11	61.1	NA	
Aged 65-74	Poor vision only	48	14.8	34	15.2	15	11.0	7	10.5	2	11.1	NA	
Aged 65-74	Poor hearing only	47	14.5	34	15.2	23	16.8	11	16.4	2	11.1	NA	
Aged 65-74	Dual sensory loss	32	9.9	26	11.6	9	6.6	4	6.0	3	16.7	NA	
Aged 75+	Good vision and hearing	97	47.8	114	55.9	108	50.5	118	53.4	88	44.9	82	49.7
Aged 75+	Poor vision only	46	22.7	19	9.3	22	10.3	25	11.3	33	16.8	18	10.9
Aged 75+	Poor hearing only	33	16.3	41	20.1	44	20.6	39	17.7	46	23.5	34	20.6
Aged 75+	Dual sensory loss	27	13.3	30	14.7	40	18.7	39	17.7	29	14.8	31	18.8
**Age**	**Sensory loss**	**Men 1994**		**Men 1996**		**Men 1998**		**Men 2000**		**Men 2002**		**Men 2004**	
All 65+	Good vision and hearing	257	55.3	173	47.4	160	53.9	118	47.2	86	48.3	69	46.9
All 65+	Poor vision only	51	11.0	29	8.0	21	7.1	17	6.8	17	9.6	13	8.8
All 65+	Poor hearing only	106	22.8	115	31.5	83	28.0	73	29.2	49	27.5	46	31.3
All 65+	Dual sensory loss	51	11.0	48	13.2	33	11.1	42	16.8	26	14.6	19	12.9
Aged 65-74	Good vision and hearing	175	55.4	118	52.4	93	61.6	44	53.0	17	54.8	NA	
Aged 65-74	Poor vision only	33	10.4	13	5.8	10	6.6	5	6.0	3	9.7	NA	
Aged 65-74	Poor hearing only	72	22.8	71	31.6	38	25.2	23	27.7	8	25.8	NA	

Aged 65-74	Dual sensory loss	36	11.4	23	10.2	10	6.6	11	13.3	3	9.7	NA	
Aged 75+	Good vision and hearing	82	55.0	55	39.3	67	45.9	74	44.3	69	46.9	69	46.9
Aged 75+	Poor vision only	18	12.1	16	11.4	11	7.5	12	7.2	14	9.5	13	8.8
Aged 75+	Poor hearing only	34	22.8	44	31.4	45	30.8	50	29.9	41	27.9	46	31.3
Aged 75+	Dual sensory loss	15	10.1	25	17.9	23	15.8	31	18.6	23	15.7	19	12.9

Data source: MELSHA survey data, no weights used.

Table S2. Persistence of DSL, hearing loss and vision loss at baseline plus 2 years, plus 6 years and plus 10 years.

MELSHA 1994-1996	Individual Characteristics	Women		Men	
**Variables**	**Values**	**n**	**%**	**n**	**%**
Persistent DSL (1994-1996)	No DSL in 1994 and 1996	351	82.6%	299	82.1%
	DSL in 1994 only	18	4.2%	18	4.2%
	DSL in 1996 only	33	7.8%	29	8.0%
	DSL in 1994 and 1996	23	5.4%	18	4.9%
	Total	425	100%	364	100%
Persistent hearing (1994-1996)	Good hearing in 1994 and 1996	273	63.9%	184	50.4%
	Poor hearing in 1994 only	24	5.6%	19	4.5%
	Poor hearing in 1996 only	52	12.2%	62	17.0%
	Poor hearing in 1994 and 1996	78	18.3%	100	27.4%
	Total	427	100%	365	100%
Persistent vision (1994-1996)	Good vision in 1994 and 1996	269	63.1%	251	68.8%
	Poor vision in 1994 only	49	11.5%	37	8.7%
	Poor vision in 1996 only	46	10.8%	37	10.1%
	Poor vision in 1994 and 1996	62	14.6%	40	11.0%
	Total	426	100%	365	100%

MELSHA 1994-2000	Individual Characteristics	Women		Men	
Variables	Values	n	%	n	%
Persistent DSL (1994-2000)	No DSL in 1994 and 2000	229	80.4%	191	76.4%
	DSL in 1994 only	13	4.6%	17	6.8%
	DSL in 2000 only	34	11.9%	32	12.8%
	DSL in 1994 and 2000	9	3.2%	10	4.0%
	Total	285	100%	250	100%
Persistent hearing (1994-2000)	Good hearing in 1994 and 2000	172	60.4%	112	44.8%
	Poor hearing in 1994 only	20	7.0%	23	9.2%
	Poor hearing in 2000 only	48	16.8%	52	20.8%
	Poor hearing in 1994 and 2000	45	15.8%	63	25.2%
	Total	285	100%	250	100%
Persistent vision (1994-2000)	Good vision in 1994 and 2000	178	62.2%	163	65.2%
	Poor vision in 1994 only	34	11.9%	28	11.2%
	Poor vision in 2000 only	39	13.6%	33	13.2%
	Poor vision in 1994 and 2000	35	12.2%	26	10.4%
	Total	286	100%	250	100%

MELSHA 1994-2004	Individual Characteristics	Women		Men	
Variables	Values	n	%	n	%
Persistent DSL (1994-2004)	No DSL in 1994 and 2004	125	76.7%	118	80.3%
	DSL in 1994 only	7	4.3%	10	6.8%
	DSL in 2004 only	26	16.0%	14	9.5%
	DSL in 1994 and 2004	5	3.1%	5	3.4%
	Total	163	100%	147	100%
Persistent hearing (1994-2004)	Good hearing in 1994 and 2004	86	52.8%	65	44.2%
	Poor hearing in 1994 only	12	7.4%	17	11.6%
	Poor hearing in 2004 only	41	25.2%	34	23.1%
	Poor hearing in 1994 and 2004	24	14.7%	31	21.1%

	Total	163	100%	147	100%
Persistent vision (1994-2004)	Good vision in 1994 and 2004	97	58.8%	99	67.4%
	Poor vision in 1994 only	19	11.5%	16	10.9%
	Poor vision in 2004 only	33	20.0%	19	12.9%
	Poor vision in 1994 and 2004	16	9.7%	13	8.8%
	Total	165	100%	147	100%

Data source: MELSHA survey data, no weights used.

Table S3 . Multivariate regression results for DSL prevalence and its associated outcomes in 1994 for women.

**Women 1994**	**DVs**	**DSL 1994**	**Unmet needs for hearing aids 1994**	**Poor/fair health**	**Depressed 1994**	**Perceived inadequate social activities 1994**	**Community services use 1994**	**Aged in place 1994-2004**
Individual characteristics		Coef.	Coef.	Coef.	Coef.	Coef.	Coef.	Coef.
Age group	Aged 65-74							
	Aged 75+	0.175	-0.155	-0.186	0.180	-0.103	1.013*	-0.432
Marital status	Current living with partner (Ref.)							
	Previously with a partner	-0.476	-0.322	0.151	0.506	0.420	0.956*	-0.370
	Never married	-1.689	-0.648	0.227	0.580	0.347	0.483	-1.430*
Living alone	No (Ref.)							
	Yes	1.243*	0.716*	-0.300	-0.008	-0.279	-0.147	0.075
Housing tenure type	Own house (Ref.)							
	Paying off house	-0.536	-0.559	-1.253	0.939	0.344	0.547	0.263
	Public tenants	-0.318	0.113	0.873*	0.256	-0.199	-0.299	-0.418
	Private renter	-0.349	-0.016	0.729	-0.378	0.000	-0.224	-2.046 *
	Other	1.123*	0.115	-2.179*	0.138	-0.927	-0.923	-0.392
Main income source	Government benefit (Ref.)							
	Private income	-1.311	-0.078	-0.103	-0.095	0.074	-0.768	-0.009
Vision and	Good vision and hearing							

hearing	(Ref.)							
	Poor vision only			1.035*	0.762*	0.257	0.353	0.590
	Poor hearing only			-0.229	0.580*	0.799*	0.957*	0.132
	Dual sensory loss			1.410*	1.237*	0.616*	0.687*	-0.199
Constant		-2.284	-1.433	-1.754	-2.299	-1.691	-2.849	0.906
R-square		0.079	0.012	0.079	0.0547	0.022	0.1087	0.062
Sample		517	517	514	517	493	488	287

Data source: MELSHA survey data, no weights used. Note: * indicates significance at the 10% level. Ref.=reference group. Coef.=estimated coefficient.

Table S4 . Multivariate regression results for DSL prevalence and its associated outcomes in 1994 for men.

**Men 1994**	**DVs**	**DSL 1994**	**Unmet needs for hearing aids 1994**	**Self-reported poor/fair health 1994**	**Depressed 1994**	**Perceived inadequate social activities 1994**	**Community services use 1994**	**Aged in place**
Individual characteristics		Coef.	Coef.	Coef.	Coef.	Coef.	Coef.	Coef.
Age group	Aged 65-74 (Ref.)							
	Aged 75+	-0.093	-0.202	-0.754*	0.094	0.193	1.314*	-1.431 *
Gender	Male (Ref.)							
	Female	0.000	0.000	0.000	0.000	0.000	0.000	0.000
Marital status	Current living with partner (Ref.)							
	Previously with a partner	0.424	-0.637	1.037*	0.790	0.709	0.723	-0.110
	Never married	0.201	-0.657	1.104*	-0.745	1.845*	2.080*	0.530
Living alone	No (Ref.)							
	Yes	0.325	0.438	-0.520	0.423	-0.751	-0.760	-0.095
Housing tenure type	Own house (Ref.)							
	Paying off house	0.863	0.726	0.875*	0.672	0.119	0.000	-0.420
	Public tenants	0.000	-0.564	-0.234	0.000	-0.071	0.000	-0.453
	Private renter	0.000	1.020*	-0.566	-0.034	-0.150	0.470	-0.285
	Other	-0.362	-0.770	0.441	0.285	0.864	-0.870	-1.007
Main income source	Government benefit (Ref.)							
	Private income	-0.566*	0.349	-0.376	0.013	0.151	-1.498*	0.183
Vision and hearing	Good vision and hearing (Ref.)							
	Poor vision			0.424	0.572	0.676*	-1.328	-0.580

	only							
	Poor hearing only			0.531*	0.855*	0.663*	0.021	-0.289
	Dual sensory loss			0.832*	0.528	1.032*	1.265*	-0.317
Constant		-2.021	-1.188	-1.687	-2.828	-2.277	-3.194	2.006
R-square		0.0293	0.0218	0.0616	0.0539	0.056	0.158	0.097
Sample		435	458	453	450	457	394	318

Data source: MELSHA survey data, no weights used. Note: * indicates significance at the 10% level. Ref.=reference group. Coef.=estimated coefficient.

Table S5 . Multivariate regression results for DSL prevalence and its associated outcomes in 1994 for men and women.

**Women and men 1994**	**DVs**	**DSL 1994**	**Unmet needs for hearing aids 1994**	**Poor/fair health1994**	**Depressed 1994**	**Perceived inadequate social activities 1994**	**Community services use 1994**	**Aged in place 1994-2004**
**Individual characteristics**		Coef.	Coef.	Coef.	Coef.	Coef.	Coef.	Coef.
Age group	Aged 65-74 (Ref.)							
	Aged 75+	0.036	-0.180	-0.414*	0.160	-0.014	1.059*	-0.899*
Gender	Male (Ref.)							
	Female	-0.324	-0.267	-0.081	0.447*	0.190	0.752*	-0.591*
Marital status	Current living with partner (Ref.)							
	Previously with a partner	-0.032	-0.389	0.453	0.585*	0.577*	0.894*	-0.207
	Never married	-0.749	-0.698	0.696	0.223	1.246*	1.005*	-0.589
Living alone	No (Ref.)							
	Yes	0.827*	0.598*	-0.319	0.113	-0.432	-0.248	0.097
Housing tenure type	Own house (Ref.)							
	Paying off house	0.468	0.268	0.291	0.760*	0.237	-0.188	-0.172
	Public tenants	-0.617	0.035	0.586	0.000	-0.125	-0.527	-0.570
	Private renter	-0.937	0.474	0.306	-0.206	-1.066*	-0.101	-0.981*
	Other	0.723	-0.080	-0.904	0.191	-0.353	-0.796	-0.470

Main income source	Government benefit (Ref.)							
	Private income	-0.868*	0.140	-0.226	-0.037	0.117	-0.978*	0.117
Vision and hearing	Good vision and hearing (Ref.)							
	Poor vision only			0.771*	0.694*	0.392	0.071	0.247
	Poor hearing only			0.196	0.702*	0.700*	0.679*	0.006
	Dual sensory loss			1.135*	0.983*	0.762*	0.808*	-0.210
Constant		-1.996	-1.145	-1.71462	-2.80738	-2.059	-3.403	1.611
R-square		0.0463	0.0105	0.044	0.064	0.030	0.133	0.074
Sample		975	975	967	975	969	911	605

Data source: MELSHA survey data, no weights used. Note: * indicates significance at the 10% level. Ref.=reference group. Coef.=estimated coefficient.

Table S6. Multivariate regression results for DSL incidence and its influences in 2004 for women.

**Women 2004**	**DVs**	**DSL 2004**	**Unmet needs for hearing aids 2004**	**Poor/fair health 2004**	**Depressed 2004**	**Perceived inadequate social activities 2004**	**Community services use 2004**	**Aged in place 1994-2010**
Individual characteristics		Coef.	Coef.	Coef.	Coef.	Coef.	Coef.	Coef.
Marital status	Current living with partner (Ref.)							
	Previously with a partner	1.457	-0.529	-0.500	0.209	1.389*	0.425	0.177
	Never married	0.299	-1.307	0.413	0.000	1.828	1.603	0.000
Living alone	No (Ref.)							
	Yes	-0.120	0.843	-0.043	-0.642	-0.686	0.653	2.205
Housing tenure type	Own house (Ref.)							
	Paying off house	0.000	0.000				0.000	0.000
	Public tenants	0.000	0.000	1.342	0.401	1.231	0.000	-5.127*
	Private renter	1.015	0.606	0.000	1.724	-0.470	0.998	0.000
	Other	2.063*	1.297	-0.334	-1.060	0.804	-1.001	0.000
Main income	Government benefit (Ref.)							

source								
	Private income	-0.162	0.028	0.997*	0.179	0.661	-0.353	-2.694*
Vision and hearing	Good vision and hearing (Ref.)							
	Poor vision only			0.128	-0.311	1.048	0.192	0.000
	Poor hearing only			0.037	-0.010	1.788*	0.845*	0.239
	Dual sensory loss			2.449*	1.374*	1.940*	1.024*	-2.175
Consstant		-2.532	-1.590	-2.194	-1.502	-3.481	-1.576	3.220
R-square		0.0899	0.0236	0.155	0.0608	0.1419	0.099	0.306
Sample		145	145	146	144	150	139	56

Data source: MELSHA survey data, no weights were used. Note: * indicates significance at the 10% level. Ref.=reference group. Coef.=estimated coefficient.

Table S7. Multivariate regression results for DSL incidence and its influences in 2004 for men.

**Men, 2004**	**DVs**	**DSL 2004**	**Unmet needs for hearing aids 2004**	**Poor/fair health 2004**	**Depression2004**	**Perceived inadequate social activities 2004**	**Community services use 2004**	**Aged in place 1994-2010**
**Individual characteristics**		Coef.	Coef.	Coef.	Coef	Coef.	Coef.	Coef.
Marital status	Current living with partner (Ref.)							
	Previously with a partner	1.348	1.014	-4.260*	1.900	1.629*	-1.889	NA
	Never married	1.783	2.653*			1.488	0.420	NA
Living alone	No (Ref.)							
	Yes	-1.376	-2.729*	2.667*	-1.966	-0.837	1.583	NA
Housing tenure type	Own house (Ref.)							
	Paying off house		2.730*	0.551		2.618		NA
	Public tenants							
	Private renter							
	Other	-0.287		0.232	-1.075	-1.334	1.883*	-1.058

Main income source	Government benefit (Ref.)							
	Private income	-0.200	-1.240*	-0.483	-0.727	-0.624	-1.442*	0.080
Vision and hearing	Good vision and hearing (Ref.)							
	Poor vision only			1.944*	1.180	1.909	-0.897	-1.052
	Poor hearing only			0.529	0.980	0.745	0.275	-0.876
	Dual sensory loss			1.371*	2.889	0.717	0.896	0.000
Constant		-1.942	-0.745	-1.907	-3.112	-2.260	-1.217	2.661
R-square		0.0251	0.1091	0.141	0.201	0.102	0.130	0.057
Sample		127	123	124	122	129	121	56

Data source: MELSHA survey data, no weights used. Note: * indicates significance at the 10% level.
Ref.=reference group. Coef.=estimated coefficient.
